# Between commitment and avoidance – working age stroke survivors’ perceptions of physical activity and sedentary behaviour: a qualitative study

**DOI:** 10.1186/s12883-022-02704-3

**Published:** 2022-05-17

**Authors:** Kirsti S. Roaldsen, Charlotte Walter, Johan Gäverth, Ing-Mari Dohrn

**Affiliations:** 1 Department of Neurobiology, Care Sciences and Society, Karolinska Institutet, SE-141 83 Huddinge, Stockholm, Sweden; 2 Department of Research, Sunnaas Rehabilitation Hospital, Nesodden, Norway; 3 Faculty of Health Sciences, Oslo Metropolitan University, Oslo, Norway; 4 Neurocampus, Stockholm, Sweden; 5 Research and Development Unit, Aleris Rehab Station Stockholm/Spinalis, Stockholm, Sweden; 6 Unit of Occupational and Physical Therapy, Södersjukhuset, Stockholm, Sweden

**Keywords:** Adults, Content analysis, Disability, Exercise, Interviews, Stroke, Young

## Abstract

**Background:**

It is critical for stroke survivors in working age to develop skills and confidence for long-term self-management of physical activity and exercise training to maintain a healthy lifestyle and decrease the risk of recurrent stroke and other cardiovascular diseases. Still, knowledge is scarce about concerns and experiences of physical activity and sedentary behaviour after stroke in working age, and further qualitative studies are required. The aim of this study was to explore and describe perceptions of physical activity and sedentary behaviour in stroke survivors under 65 years who are living with disability.

**Methods:**

A qualitative design with individual semi-structured interviews was selected to generate rich data. Ten informants aged 36–61 years were interviewed 0.5–25 years after their stroke. The interviews were analysed with qualitative content analysis, with an inductive and interpretive approach.

**Results:**

A two-sided contradictory relationship to physical activity and sedentary behaviour was identified. The overarching theme found was “Physical activity and sedentary behaviour – between commitment and avoidance”, comprising three main themes; “Physical activity – medicine for body and mind”, “Physical activity reminds of limitations”, and “Sedentary behaviour – risk, rest, and alternative”. The informants perceived physical activity as medicine, important for both physical and mental functioning, but also as a constant reminder of having a body that no longer functions as it used to. These mixed perceptions and feelings influenced the informants’ behaviours related to physical activity and sedentary behaviour, and both commitment and avoidance were clear strategies.

**Conclusions:**

Working age stroke survivors expressed a clear positive perception of the importance of physical activity for health. However, physical activity was also described as a strong reminder of limitations which paradoxically could lead to sedentary behaviour. To support a physically active lifestyle post stroke, effective interventions as well as health promotion, counselling and patient education are imperative. These should be delivered by appropriately skilled health care professionals.

## Background

Even though the incidence of first-time stroke has decreased, stroke in working age adults (< 65 years) has increased [1, 2]. In 2020, about 25,400 persons in Sweden were diagnosed with stroke and 20% of those were under 65 years, including 4% under 50 [1, 2]. Around 13% of stroke survivors under the age of 50 have a remaining disability, affecting the ability to function independently [3], with large consequences for persons in working-age [4].

In 2020 the World Health Organization released the first guidelines for people living with disability emphasising the health benefits of physical activity and limiting sedentary behaviour [5]. Physical activity and exercise are essential to reduce disability and improve fitness, walking capacity and balance after stroke [5, 6], yet underused components of poststroke care [7]. Recent studies have shown that community-dwelling stroke survivors are more sedentary than age-matched healthy peers and many stroke survivors do not meet the physical activity recommendations [8, 9]. This may increase the risk of recurrent stroke and other cardiovascular diseases [7]. It is therefore critical for younger stroke survivors to develop skills and confidence for long-term self-management of physical activity and exercise training. For effective health promotion and counselling, health professionals must meet the needs and preferences of this patient group. Still, knowledge is scarce about concerns and experiences of physical activity and sedentary behaviour after stroke in working age adults [10] and further qualitative studies are required. The aim of this study was to explore and describe perceptions of physical activity and sedentary behaviour in stroke survivors under 65 years who are living with disability.

## Methods

### Informants

Ten informants, 6 women and 4 men, were purposely recruited from the Swedish Stroke Association (Stockholm District, Stroke in the middle of life (SMILE)) through Aleris Rehab Station Stockholm/Spinalis, and from three physiotherapy clinics in Stockholm. Inclusion criteria were: age 18–65 years, at least 6 months after onset of stroke, remaining physical and/or mild cognitive disability, Swedish speaking, and without apparent speech or language difficulties. The strategy was to achieve a diversity in sex, age, and physical or cognitive function. For background characteristics see Table 1.

**Table 1:** Demographic characteristics of informants, *n* = 10

**Age, years**	56.5 (36–61)
**Sex, women / men**	6 / 4
**Time since stroke**	
< 1 year	2
1–3 years	3
8–24 years	5
**Living status**	
alone	6
alone with children	2
with partner	2
**Working status**	
working part-time	3
on sick leave	4
on disability pension	3
**Ambulatory status**^**a**^	
walking without aid	7
walking without aid indoors, motorised wheelchair outdoors	1
walking with aid	2
**Activities of daily living**	
independent	8
with home-care services	2
**Self-reported physical activity**^**b**^**, hours/week**	
low intensity	7 (1–15)
moderate-to-vigorous intensity	1.8 (0–4.5)

^a^at least 75% of the time

^b^low intensity: e.g., walking, household chores, light gardening; moderate-to-vigorous intensity: e.g., aerobic exercise, cycling, water exercise, running

Data are presented as numbers or median (range)

The study was conducted according to the Declaration of Helsinki and each informant signed an informed consent before participation. Oral and written information of the study purpose and the right to withdraw at any time without given reason were given to the informants both at recruitment and before the interview. The informants were assured about the confidentiality of the recorded conversations and that they would not be named in the final description and analysis. The study was approved by the Chief physician, Aleris Rehab Station Stockholm/Spinalis, 14- 05-10.

### Data collection

Individual interviews were selected for the capacity to generate rich data [11], and a semi-structured interview guide with open-ended questions was developed to allow informants the freedom to express their views in their own words [11]. The guide covered the topics: ‘To live with the effects of a stroke’, ‘The concept of physical activity’, ‘Possibilities’, ‘Challenges’ and ‘Advice to others’. A set of follow-up questions, such as: ‘Tell me more about … ’ and ‘Can you give an example of …? ’ was also included in the guide. ‘Physical activity’ was consistently used and no specific questions about ‘exercise’ or sedentary behaviour’ were included in the guide. However, when ‘exercising’ or ‘sitting/being sedentary’ were mentioned by the informants the interviewer used these concepts in the follow-up questions. Before ending the interview, the informants were asked if there was anything more they would like to add. Prompts, such as repeating the informant’s words, summarising the main idea, or expressing interest with verbal agreement, were used to encourage the informants to continue talking and to elicit more details. Two pilot interviews were conducted to test the guide, resulting only in addition of a few follow-up questions, and the pilot interviews were therefore included in the study. The interviews took place at locations selected by the informants, i.e., at the informants’ workplace or home, a physiotherapy clinic or at a quiet café, during the spring of 2015. All interviews were conducted face-to-face by one of the authors (CW), a physiotherapist with several years of experience from interprofessional stroke rehabilitation, mainly for patients in working-age. The experience of meeting with persons with stroke, allowed the interviewer to respond to questions or concerns that could arise during the interview, which ensured the protection of the informants. Persons with previous, ongoing, or planned care contact with the interviewer were excluded from the study. The interviews lasted 35–55 minutes and the recordings were transcribed verbatim. Background characteristics were collected with a brief study specific questionnaire.

### Data analysis

A qualitative content analysis with an inductive approach was used to identify and interpret codes and themes related to physical activity and sedentary behaviour [12, 13]. The analysis process followed the criteria for credibility, dependability, and transferability, described by Elo et al. [14], to ensure the quality of the findings and trustworthiness of the interpretations.

All authors were involved in the analysis process, and the authors’ combined clinical and research experience was complementary and of importance to the analysis. KSR is physiotherapist researcher with extensive experience of qualitative research and neurological rehabilitation, CW is physiotherapist with long experience from stroke rehabilitation, JG is researcher and physiotherapist with specialist competence in neurology, and IMD is physiotherapist researcher with experience of qualitative research and expertise in physical activity and sedentary behaviour.

The rigorous and systematic analysis process was carried out in several steps [13, 14]: reading of transcripts for familiarization with the data; identifying all meaning units corresponding to the aim; condensation and generating initial codes; searching for subthemes and themes to interpret the underlying meaning. Finally, an overarching theme was identified. The process involved going back and forth between the different steps to capture the key aspects in the transcripts, and the findings were repeatedly discussed by the authors until consensus was reached. Examples of the steps are presented in Table 2. Both the interviews and the data analysis process were performed in Swedish. The citations were translated and reviewed by a bilingual physiotherapist.

**Table 2:** Examples of the steps in the analysis process

Meaning unit	Condensed text	Code	Subtheme	Main theme
“With exercise I can make the pain go away, or at least reduce it. That’s why I avoid a lot of painkillers.”	Prefer exercise instead of painkillers	Physical activity reduces pain	Physical activity is treatment	Physical activity – medicine for body and mind
“I tried to ride a bike last autumn but I haven’t tried again now this spring. I don’t think I dare. If something happens unexpectedly, like a noise or someone appearing suddenly, my reaction is very strong.”	I don’t dare to ride a bicycle anymore because something unexpected might happen	Fear of falling	Feelings of insecurity	Physical activity reminds of limitations
“In the beginning when I couldn’t sit up straight, I thought I’ll never be able to go outside … I’ll be like an old person, just sit [in the wheelchair] and look out the window. That’s how I was in the beginning. But now, I hardly sit at all, I don’t sit all day.”	I was afraid I would be sitting and not be able walk again, but now I hardly sit at all	Avoid wheelchair	Risk factor for impairment and dependence	Sedentary behaviour – risk, rest, and alternative

## Results

The analysis resulted in one overarching theme, comprising three main themes and 11 subthemes (Table 3).

**Table 3:** Overview of the results: Overarching theme, main themes, and subthemes

Overarching theme	Main themes	Subthemes
Physical activity and sedentary behaviour – between commitment and avoidance	Physical activity – medicine for body and mind	Physical activity is treatment
		Strengthened identity and self-image
		Exercising gives a sense of context and meaning
		Positive emotions and well-being
	Physical activity reminds of limitations	Demands and frustration
		Reminder of disability
		Feelings of insecurity
		Increased pain and discomfort
	Sedentary behaviour – risk, rest, and alternative	Risk factor for impairment and dependence
		Necessary for recovery
		Sedentary activities for body and mind

### Overarching theme: Physical activity and sedentary behaviour – between commitment and avoidance

The overarching theme was identified from the two-sided contradictory relationship to physical activity and sedentary behaviour revealed in the interviews. The informants perceived physical activity as medicine, important for both physical and mental functioning, but also as a constant reminder of having a body that no longer functions as it used to. The relationship between these two different sides varied. For some informants, positive experiences dominated, while others described frustration or sadness. They also mentioned how they were torn between these two perceptions, which gave them a guilty conscience. Sedentary behaviours were also mentioned in two contradictory contexts. First, as a risk factor necessary to avoid, to be able to function and stay healthy, closely related the perception of physical activity as medicine. And on the other hand, as a chance to rest and recover after being active, or as an alternative way to be active physically or mentally. These mixed perceptions and feelings influenced the informants’ behaviours related to physical activity and sedentary behaviour, and both commitment and avoidance were clear strategies.

### Main theme 1: Physical activity – medicine for body and mind

The first main theme summarises the perception of physical activity as healing and health enhancing. A way forward to increased well-being and improved body functions, which enables participation. The informants described both immediate effects, such as feelings of joy, contentment and confirmation, and more lasting effects, such as better sleep and pain relief. In a broader perspective, physical activity was described as giving meaning in life and a sense of context, linked to self-image and identity.

*“With exercise, something happens, even if you don’t believe it. We will never be like before, that’s the way it is, but we will be much better.”* (I6)

### Physical activity is treatment

Some described physical activity as their way to return or come closer to the life before the stroke. Physical activity was perceived as medicine, with positive effects on both physical and mental functions, but without negative side effects.

*“Physical activity is the only thing that can make me, let’s say, become normal. Return to my normal life. And there are no other ways, no other pills, no such thing.”* (I9)

### Strengthened identity and self-image

It emerged that physical activity gave purpose to life and was essential for the daily life to work. It was a confirmation of things they could do and gave courage to try new things. Being able to strive forward and to set and achieve goals led to increased self-confidence.

*“ I notice that I can do more for myself now. Going to the gym and exercising on my own, that also increases my self-confidence.”* (I3)

### Exercising gives a sense of context and meaning

The informants talked about the lack of context when friends disappear or when abilities are lost. When work or previous interests cannot be performed anymore. Being able to exercise again could fill that emptiness. Having a plan for the day and reason to get out of the house and socialise with other people, meant a lot. Exercising with peers gave valuable interactions and opportunities to find new friends and get inspired. Regular exercising was also seen as a job and a strategy to motivate oneself to continue day after day, and to take responsibility for making the best of the situation.

*“I don’t say “I’m going to exercise now”, I say “I’m going to work” - this is my job. I walk and I cycle, and do this and that and the other. That’s the way it is. We work with our own body – it’s also a job. That’s why we have … those who have sickness benefits or such, it’s also a job they have. You have to work with yourself.”* (I6)

### Positive emotions and well-being

Physical activity evoked positive emotions, both direct effects, such as joy and satisfaction, and more long-lasting effects on well-being. Knowledge of these positive emotions were important for motivation and their experience of physical activity.

*“Physical activity gives me harmony. It makes you happy, you know, the endorphins are bubbling, and it makes you very positive.”* (I4)

Some expressed strong emotions of gratitude for what the body can accomplish and be able to perform physical activities after a stroke. Finding forms of activity that highlight one’s resources and make the body feel and function as before gave a feeling of freedom. To be able to continue with the same activities as before the stroke, even though it was with adaptions, was appreciated. Ball games of various kinds, swimming, dance, and rhythmic training were given as examples of activities that were fun.

*“I think swimming was the first time that I felt a little normal and could move properly and almost run, stand on one leg and things like that … it was a fantastic feeling!”* (I8)

### Main theme 2: Physical activity reminds of limitations

The second main theme summarises how physical activity was a reminder of limitations and having a body that does not function as before. The limitations mentioned were both physical, cognitive, emotional, and social. Disabilities, activity limitations and participation restrictions were described as grief, loss, and reminder. Persistent symptoms after the stroke, such as mobility limitations and fatigue, lead to different needs for adaptation, planning, assistance, and logistics that affected both how physical activity was performed and experienced. The body’s limitations evoke various emotions such as frustration, fear, and bad conscience. Informants described a constant struggle that never ends. Direct consequences were pain and discomfort related to physical activity, which for some affected the experience negatively.

*“I’m still scared someone is going to bump into me and knock me over.”* (I8)

### Demands and frustration

Physical activity was also experienced as boring and monotonous, as well as difficult and exhausting. Exercising with a body that does not work at all as before felt difficult and overwhelming. Mental fatigue after being in busy environments was a perceived limitation. The new situation with everyday activities that take longer time, are more energy demanding, and require a high level of concentration generated a resistance to exercising.

*“Everyday life still takes a lot of time. If, for example, I vacuum one day and clean the kitchen, then it is almost a day’s project in addition to cooking and things you do every day. It will be, like, the event of the day.”* (I7)

Informants talked about a constant struggle, with demands both from themselves and from family members to be more physically active. Not being able or able to live up to these expectations gave a bad conscience.

### Reminder of disability

Informants expressed sadness and loss over lost bodily functions and how physical activity was a reminder of this. Lost abilities could also evoke feeling of shame and some explained how they felt uncomfortable and did not want to be judged or misinterpreted in their old environment.

*“I have been thinking about horse riding again, maybe in the future, maybe tomorrow... But I don’t want the others in the stable to be there because I know everyone watches each other … that's the way it is …"* (I4)

It was mentioned that being active requires personal responsibility and discipline, which may be difficult with cognitive dysfunction. Planning, encouragement, and help from other people, as well as adaptation of training and premises were needed. Some expressed that all the logistics related to physical activity simply had become too difficult and complicated. The need for guidance and support to be physically active could persist long after the stroke and limit the choice of activities.

### Feelings of insecurity

A fear in connection with activities, based on concerns on how the body would react or function in different situations was described. Unsecure situations mentioned were fear of falling in an escalator and not being able to tie a loose shoelace while out walking. Thoughts of exercise as a potential risk for a new stroke or risks related to other associated diseases, such as heart problems were also expressed. Living with this uncertainty was perceived as difficult to deal with.

*“I would rather exercise at a [physiotherapy] clinic than at a regular gym. I feel safe and there is a physiotherapist who is there and helps all the time. If I start doing something [wrong], she says no, no, no … (laughs) … She has a watchful eye all the time and she knows what we need to do.”* (I1)

### Increased pain and discomfort

It was reported that physical activity could cause increased pain and discomfort, not only during the activity itself, but also lasting for a longer period. This led to a reluctance, which was handled in different ways, either through decreased activity or by adapting the activity to make it work. Some informants described how the pain and discomfort negatively affected the whole experience of physical activity.

*“So, I have pain, but it will be twice as much if I take my stick and walk. I like to walk and go out and do things like that, but I can’t. It's not that I don’t dare … but I know I will get a lot of pain afterwards.”* (I6)

### Main theme 3: Sedentary behaviour – risk, rest, and alternative

The third main theme summarises a multifaceted view of sedentary behaviour. It was described both as a risk factor and a chance to recover. The informants expressed how it was essential to avoid sedentary behaviour to regain independence and to stay healthy and ambulatory. But they also described how it could enable meaningful activities and how they needed to rest both physically and mentally, especially after being active.

*“I have to think about myself, that I must push myself, I know it's not good if I’m lazy and just sit.”* (I4)

### Risk factor for impairment and dependence

Several informants expressed a fear of being dependent of a wheelchair and how that was a strong motivator for being active and avoid sedentary behaviour. They talked about how it was essential to get up from the chair to be able to walk and how sedentary behaviour was a health risk in general. It was also mentioned that being sedentary affected their mood negatively.

*“No, you have to make an effort. And you can’t just sit in front of the TV. It’s easy to get stuck there [ … ] The worst thing you can do is stay in bed and think that help will come to you.”* (I1)

### Necessary for recovery

Some informants were affected by fatigue that influenced their everyday life and described how they needed to be sedentary after being active. To schedule time for rest was a strategy to still be able to work and exercise.

*“It’s not that I'm so tired that I can’t do anything. But that’s how it is right after … but with that said, I recover quite fast. But it will have an impact on the rest of the day.”* (I10)

### Sedentary activities for body and mind

Different sedentary activities, such as computer use, playing bridge, or performing exercises in sitting position, were also mentioned as meaningful activities with a possibility to challenge both mental and body functions. The first small steps on the way to be physically active again were perceived as very important in the rehabilitation process.

*“In the beginning when I started here [at the tennis club], I actually started sitting on a chair. So, I sat on a regular chair at the baseline and then the coaches hit the balls so I could reach. Well, I can only say that these guys down here have been great!”* (I2)

## Discussion

The novelty of this study is that it explores the experiences and perceptions of physical activity and sedentary behaviour in working age stroke survivors, an increasing group. The analysis yielded an overarching theme: *Physical activity and sedentary behaviour – between commitment and avoidance*. It was clear how the informants struggled with the insight that physical activity was essential to regain and retain psychical function, and the perceived difficulties and challenges with a body that did not work as before. They also expressed how important it was to avoid a sedentary lifestyle, but at the same time they experienced an increased need to rest. Physical activity became a reminder of problems and difficulties due to their disability after the stroke and this was reflected in feelings of frustration and sadness. On the other hand, we found that engaging in physical activity was perceived as a possible way to normality and independence. These findings are in line with similar research exploring perspectives of stroke survivors in working age [10, 15], describing the struggle to balance priorities between sitting and moving. The awareness of shortcomings related to difficulties to perform tasks due to lost body functions described by our informants has previously been reported to decrease quality of life in stroke survivors [15]. Whereas enriching social relations, resumption of activities, successful return to work, and continuity and presence of professional support during the rehabilitation have been reported as important factors that increase quality of life [15].

Finding social, meaningful, self-selected activities already during rehabilitation has been shown to be important [16]. The informants expressed their belief in physical activity as treatment and how their actions could have an impact on their future health, which motivated them to continue. The importance of having purpose in everyday life also yielded, and that exercise can become a substitute for work, which is in line with previous findings [17]. Enjoyment, belief in positive health effects of physical activity, as well as perceived psychological and emotional effects, are important motivating factors for stroke survivors to engage in and maintain exercising [18, 19].

It should be noted that stroke survivors, caregivers and physiotherapists may have different views on goals and interventions. According to a previous study, patients wanted activities that made them feel like the person they were before, or activities where they could develop their new identity [18]. Physiotherapists focused more on functional training and sometimes thought that their patients had unrealistic goals [18]. This highlights the importance involving patients in realistic goal setting and choice of activities to increase confidence own abilities [20]. Consequently, individuals with low confidence in their abilities and those who mainly choose avoidance as a coping strategy need to be identified by health care and rehabilitation professionals and offered increased support to be physically active.

In contrast to other studies reporting a limited awareness of the potential health consequences of sedentary behaviour among stroke survivors [21, 22], the health risks with prolonged sitting were known and stressed by the majority of the informants. Walking ability is an important goal for persons with stroke [10, 23], and one of the strongest motivators to avoid a sedentary lifestyle mentioned was to be able to walk and not use a wheelchair. Even so, some of the participants described how fatigue strongly influenced their ability to be active. To be sedentary and rest in periods was a necessary coping strategy. Similar findings have been reported from interviews with persons who recently experienced a stroke [10, 15, 22]. However, research suggests that physical activity, even at low intensity, may help lessen fatigue [24, 25]. Information and support, including learning strategies to cope with fatigue, are therefore important to guide planning of daily physical activity. The immediate post-hospital period has been suggested to be a critical time to intervene to reduce sedentary behaviour, since the mindset of some stroke survivors when leaving the hospital is to go home, rest, and recuperate [21]. Concise and user-friendly information is needed, ideally incorporating evidence-based guidance on how upright behaviours may increase well-being and reduce future stroke risk [21, 22].

In the questionnaire the informants were asked to estimate weekly time spent in physical activity at different intensities, which is known to have low agreement with device-based measurements, and this should be considered. It would be interesting to use movements sensors to get a more objective picture of physical activity and sedentary behaviour in this patient group, and to explore how the perceptions of their habits mirror the results. mHealth devices, such as smartphone applications for step count, also facilitate self-monitoring and can be used for more effective health promotion in stroke survivors [26]. Future research should explore the use of these technologies to make stroke survivors more aware of their sedentary behaviour and motivated to increase physical activity.

The results of this study reflect the perceptions of stroke survivors between 36 and 61 years, with a functional walking ability, living in an urban area in Sweden. Attitudes, cultural differences, and structural differences in health care may limit some results to be context specific and thereby limit the transferability to younger persons or those with other cultural or socioeconomic backgrounds. To further increase the understanding of physical activity and sedentary behaviour in this patient group, future research should also include informants under 36 years. The life situation at that age may look different, with more focus on studies, career, and family life with young children, which can be a difficult combination with disability after a stroke [4]. Informants living in rural areas, and with a more pronounced disabilities should also be included.

Health promotion, including counselling and patient education, is an important task for physiotherapists and especially physical activity promotion is part of the daily clinical work. All authors had clinical experience from rehabilitation and the interviewer (CW) had specific experience from rehabilitation of working age stroke survivors. This was a strength both for the data collection and in the analysis process, as the interpretation of data is influenced by the researcher’s experiences, qualifications, and training [11]. Nevertheless, prior understanding could also overshadow new meanings and hinder noticing everything in the data.

An advantage of semi-structured interviews is the richness of the collected data and such data need to be coded and interpreted in a valid and reliable way [14]. To ensure trustworthiness, the methods and results, including quotations, were described in detail according to recommendations for consolidating criteria to report qualitative interviews (COREQ) [27]. The informants represented different ages, sex, and functional status groups, which supports that a heterogenic sample was reached. This increases the credibility of the study. For dependability, the transcriptions were reviewed several times, they were checked and coded by all authors, and the interpretations were based on consensus among all authors. Further, the transparency of the analysis process, including quotations from the interviews, allows the reader to judge the trustworthiness of the results.

### Implications for practice

Our findings increase the understanding of how working age stroke survivors with disabilities experience the effects of physical activity, and how their beliefs and perceptions influence their behaviour. This knowledge can guide interventions and efforts to promote a healthy lifestyle in this target group. Individually tailored information about the positive effects of physical activity and how to cope with fatigue are important to support activity. The health risks with prolonged sitting should be highlighted, and exchanging time spent sedentary with low intensity physical activity should be encouraged. Individuals with low confidence in their abilities and those who mainly choose avoidance as a coping strategy need to be identified and offered increased support by health care professionals with expertise in health promotion, person-centered counselling, and patient education. Stroke survivors with remaining disabilities may need to be given opportunity to exercise in a quiet environment where they feel safe. Some of them may need long-term, perhaps lifelong, support from physiotherapists or other rehabilitation staff, which today is a challenge for health care. This could be facilitated through increased self-management skills as well as expanded collaborations between health care and physical activity organisers such as sports clubs and fitness centres.

## Conclusions

This study shows that the increasing population of working age stroke survivors have a clear positive perception of physical activity as medicine for body and mind. It also highlights that physical activity can be a strong reminder of limitations, which paradoxically may lead to sedentary behaviour. To support a physically active lifestyle post stroke, effective interventions as well as health promotion, counselling and patient education are imperative. These interventions should be delivered by appropriately skilled health care professionals.

## Data Availability

The data from this study are not publicly available due to the fact that the informants only provided informed consent for use of the data for the current study.
